# Supplementary material for: Content-rich biological network constructed by mining PubMed abstracts
Source: BMC Bioinformatics. 2004 Oct 8;5:147. doi: 10.1186/1471-2105-5-147 (PMC528731; doi:10.1186/1471-2105-5-147)
Supplement: Additional File 5 — The original Chilibot query results of the term "long-term potentiation (LTP)" and 22 other terms, limiting the latest references analyzed to the years 1990, 1995, 2000, and 2004. [file 1471-2105-5-147-S5.bz2 › chilibotAdditionalFile5/ltp2004/html/ZIF268_SYNAPTOTAGMIN.html]

 


 **ZIF268** and **SYNAPTOTAGMIN** 
  
Found 1 abstracts in PubMed,  **1 abstracts were retrieved and analyzed**.  


---

 Search Google  |
 PDF files only 
|  EDU domain only 

---

- J Neurosci, 2003   **Selectively reduced expression of synaptic plasticity related genes in amyloid precursor protein presenilin 1 transgenic mice.** .
  A critical question in Alzheimer s disease AD research is the cause of memory loss that leads to dementia.
  The amyloid precursor protein presenilin 1 APP PS1 transgenic mouse is a model for amyloid deposition, and like AD, the mice develop memory deficits as amyloid deposits accumulate.
  We profiled gene expression in these transgenic mice by microarray and quantitative RT PCR qRT PCR .
  At the age when these animals developed cognitive dysfunction, they had reduced mRNA expression of several genes essential for long term potentiation and memory formation Arc, **Zif268**, NR2B, GluR1, Homer 1a, Nur77 TR3 .
  These changes appeared to be related to amyloid deposition, because mRNA expression was unchanged in the regions that did not accumulate amyloid.
  Transgene expression was similar in both amyloid containing and amyloid free regions of the brain.
  Interestingly, these changes occurred without apparent changes in synaptic structure, because a number of presynaptic marker mRNAs growth associated protein 43, synapsin, synaptophysin, synaptopodin, **synaptotagmin**, syntaxin remained stable.
  Additionally, a number of genes related to inflammation were elevated in transgenic mice, primarily in the regions containing amyloid.
  In AD cortical tissue, the same memory associated genes were downregulated.
  However, all synaptic and neuronal transcripts were reduced, implying that the loss of neurons and synapses contributed to these changes.
  We conclude that reduced expression of selected genes associated with memory consolidation are linked to memory loss in both circumstances.
  This suggests that the memory loss in APP PS1 transgenic mice may model the early memory dysfunction in AD before the degeneration of synapses and neurons.
